# The genome sequence of the common green lacewing, *Chrysoperla carnea *(Stephens, 1836)

**DOI:** 10.12688/wellcomeopenres.17455.1

**Published:** 2021-12-09

**Authors:** Liam M. Crowley, University of Oxford and Wytham Woods Genome Acquisition Lab, Darwin Tree of Life Barcoding collective, Wellcome Sanger Institute Tree of Life programme, Wellcome Sanger Institute Scientific Operations: DNA Pipelines collective, Tree of Life Core Informatics collective, Darwin Tree of Life Consortium

**Affiliations:** 1 Department of Zoology, University of Oxford, Oxford, UK

**Keywords:** Chrysoperla carnea, common green lacewing, genome sequence, chromosomal, Chrysopidae

## Abstract

We present a genome assembly from an individual female *Chrysoperla carnea *(a common green lacewing; Arthropoda; Insecta; Neuroptera; Chrysopidae). The genome sequence is 560 megabases in span. The majority of the assembly (95.70%) is scaffolded into six chromosomal pseudomolecules, with the X sex chromosome assembled. Gene annotation of this assembly by the NCBI Eukaryotic Genome Annotation Pipeline has identified 12,985 protein coding genes.

## Species taxonomy

Eukaryota; Metazoa; Ecdysozoa; Arthropoda; Hexapoda; Insecta; Pterygota; Neoptera; Endopterygota; Neuroptera; Hemerobiiformia; Chrysopidae; Chrysopinae; Chrysoperla; *Chrysoperla carnea* (Stephens, 1836) (NCBI:txid189513).

## Background

*Chrysoperla carnea*, a common green lacewing, is a common and widespread lacewing across the Holarctic. It is one of the most common species of lacewing in the UK, found across a wide range of habitats. This species is part of a species complex that contains several cryptic species. These species can be distinguished by differences in the substrate-borne songs produced by adults via abdominal vibrations ( Henry *et al*., 2002). In the UK the *C. carnea* group is currently split into two species, *C. carnea* sensu stricto and *C. lucasina,* both of which appear to be common. A third species, *Chrysoperla pallida*, may also be present. *Chrysoperla carnea* overwinters as an adult in common with all *Chrysoperla* species, but has the unique trait of losing its green pigment and turning yellow-brown during the winter period. The larvae are voracious generalist predators, feeding on aphids and other insects ( Rosenheim *et al.*, 1999), including several other pest groups such as spider mites, thrips, whitefly, leafhoppers, psyllids and Lepidoptera. They have been used extensively as biocontrol agents in agricultural and horticultural systems and are commercially produced for this purpose. Adults visit flowers and feed on pollen and nectar. Females have been recorded consuming more pollen than males ( Villenave *et al*., 2005). The eggs are laid on vegetation and are suspended off the surface on characteristic stalks.

## Genome sequence report

The genome was sequenced from one male *C. carnea* collected from Wytham Woods, Oxfordshire (biological vice-county: Berkshire), UK (latitude 51.772, longitude -1.338). A total of 40-fold coverage in Pacific Biosciences single-molecule long reads and 147-fold coverage in 10X Genomics read clouds were generated. Primary assembly contigs were scaffolded with chromosome conformation Hi-C data. Manual assembly curation corrected 20 missing/misjoins and removed 6 haplotypic duplications, reducing the assembly size by 5.41% and the scaffold number by 2.32% and increasing the scaffold N50 by 0.55%.

**Figure 1.**
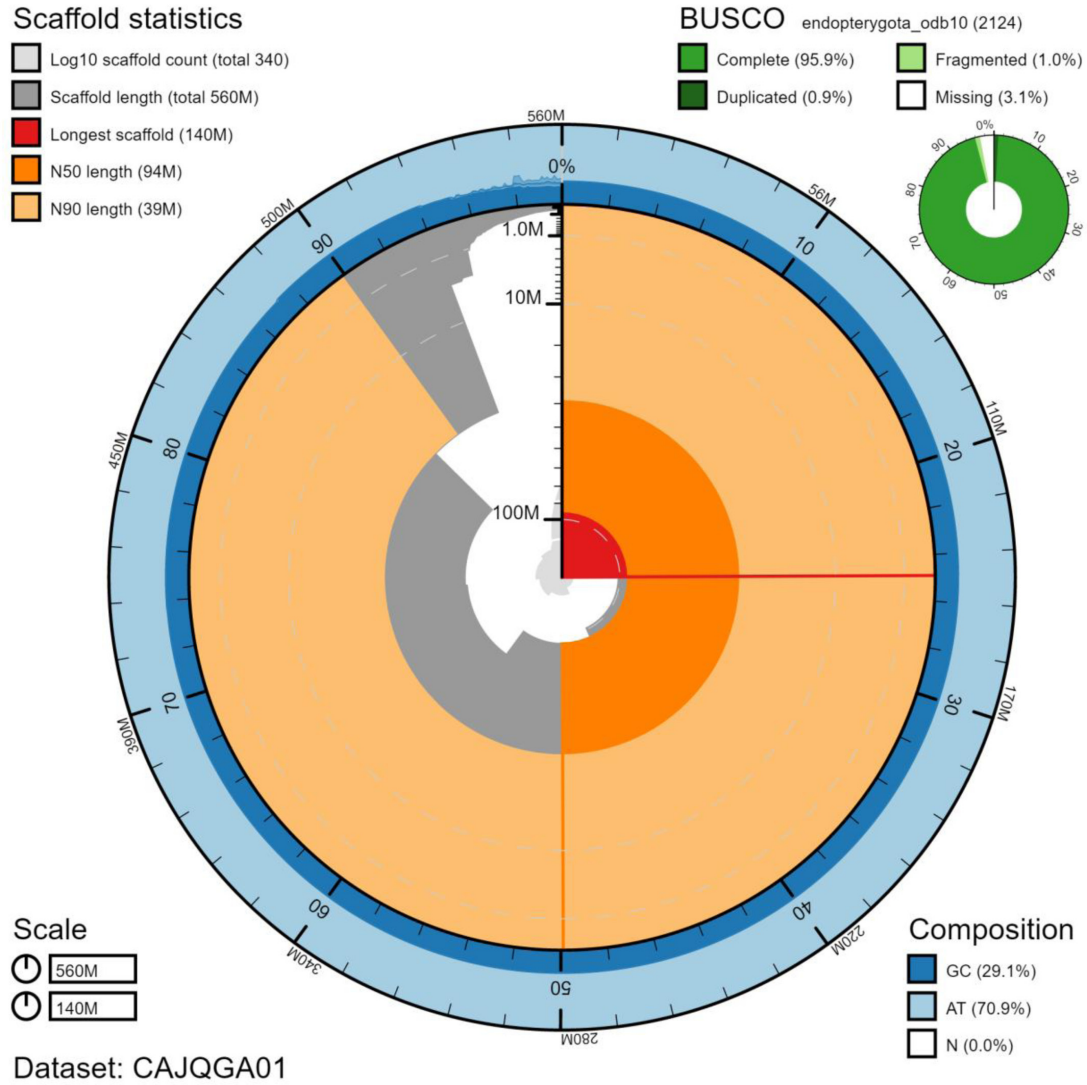
Genome assembly of *Chrysoperla carnea*, inChrCarn1.1: metrics. The BlobToolKit Snailplot shows N50 metrics and BUSCO gene completeness. The main plot is divided into 1,000 size-ordered bins around the circumference with each bin representing 0.1% of the 560,248,701 bp assembly. The distribution of scaffold lengths is shown in dark grey with the plot radius scaled to the longest scaffold present in the assembly (139,979,878 bp, shown in red). Orange and pale-orange arcs show the N50 and N90 scaffold lengths (94,407,144 and 38,618,709 bp), respectively. The pale grey spiral shows the cumulative scaffold count on a log scale with white scale lines showing successive orders of magnitude. The blue and pale-blue area around the outside of the plot shows the distribution of GC, AT and N percentages in the same bins as the inner plot. A summary of complete, fragmented, duplicated and missing BUSCO genes in the endopterygota_odb10 set is shown in the top right. An interactive version of this figure is available at https://blobtoolkit.genomehubs.org/view/inChrCarn1.1/dataset/CAJQGA01/snail.

The final assembly has a total length of 560 Mb in 337 sequence scaffolds with a scaffold N50 of 94.4 Mb ( Table 1). The majority of the assembly sequence (95.70%) was assigned to six chromosomal-level scaffolds, representing five autosomes (numbered by sequence length), and the X sex chromosome ( Figure 1– Figure 4; Table 2). There is a very large repeat associated with the X chromosome, which has resulted in the presence of many unlocalised scaffolds in the assembly. The assembly has a BUSCO v5.1.2 ( Manni *et al*., 2021) completeness of 95.9% (single 95.0%, duplicated 0.9%) using the endopterygota_odb10 reference set. While not fully phased, the assembly deposited is of one haplotype. Contigs corresponding to the second haplotype have also been deposited.

**Table 1.** Genome data for *Chrysoperla carnea*, inChrCarn1.1.

*Project accession data*	
Assembly identifier	inChrCarn1.1
Species	*Chrysoperla carnea*
Specimen	inChrCarn1
NCBI taxonomy ID	NCBI:txid189513
BioProject	PRJEB43807
BioSample ID	SAMEA7520372
Isolate information	Female, whole organism
*Raw data accessions*	
PacificBiosciences SEQUEL II	ERR6406208
10X Genomics Illumina	ERR6054664-ERR6054667
Hi-C Illumina	ERR6054668
*Genome assembly*	
Assembly accession	GCA_905475395.1
Accession of alternate haplotype	GCA_905475295.1.
Span (Mb)	560
Number of contigs	399
Contig N50 length (Mb)	67.8
Number of scaffolds	337
Scaffold N50 length (Mb)	94.4
Longest scaffold (Mb)	140
BUSCO genome score *	C:95.9%[S:95.0%,D:0.9%],F:1.0%,M:3.1%,n:2124
*Genome annotation*	
Number of genes	15,736
Number of protein-coding genes	12,985
Average length of gene (bp)	18,136
Average number of exons per transcript	4.07
Average exon size (bp)	337
Average intron size (bp)	4,304
BUSCO annotation score **	C:96.9%[S:96%,D:0.9%],F:0.2%,M:2.8%,n:2124

C= complete [S= single copy, D=duplicated], F=fragmented, M=missing, n=number of orthologues in comparison. *BUSCO scores based on the endopterygota_odb10 BUSCO set using v5.1.2, run on the inChrCarn1.1 genome assembly using BlobToolKit. A full set of BUSCO scores is available at https://blobtoolkit.genomehubs.org/view/inChrCarn1.1/dataset/CAJQGA01/busco. **BUSCO scores based on the endopterygota_odb10 BUSCO set using v4.1.4, run on the NCBI RefSeq annotation of the inChrCarn1.1 genome assembly ( NCBI *Chrysoperla carnea* Annotation Release 100).

**Figure 2.**
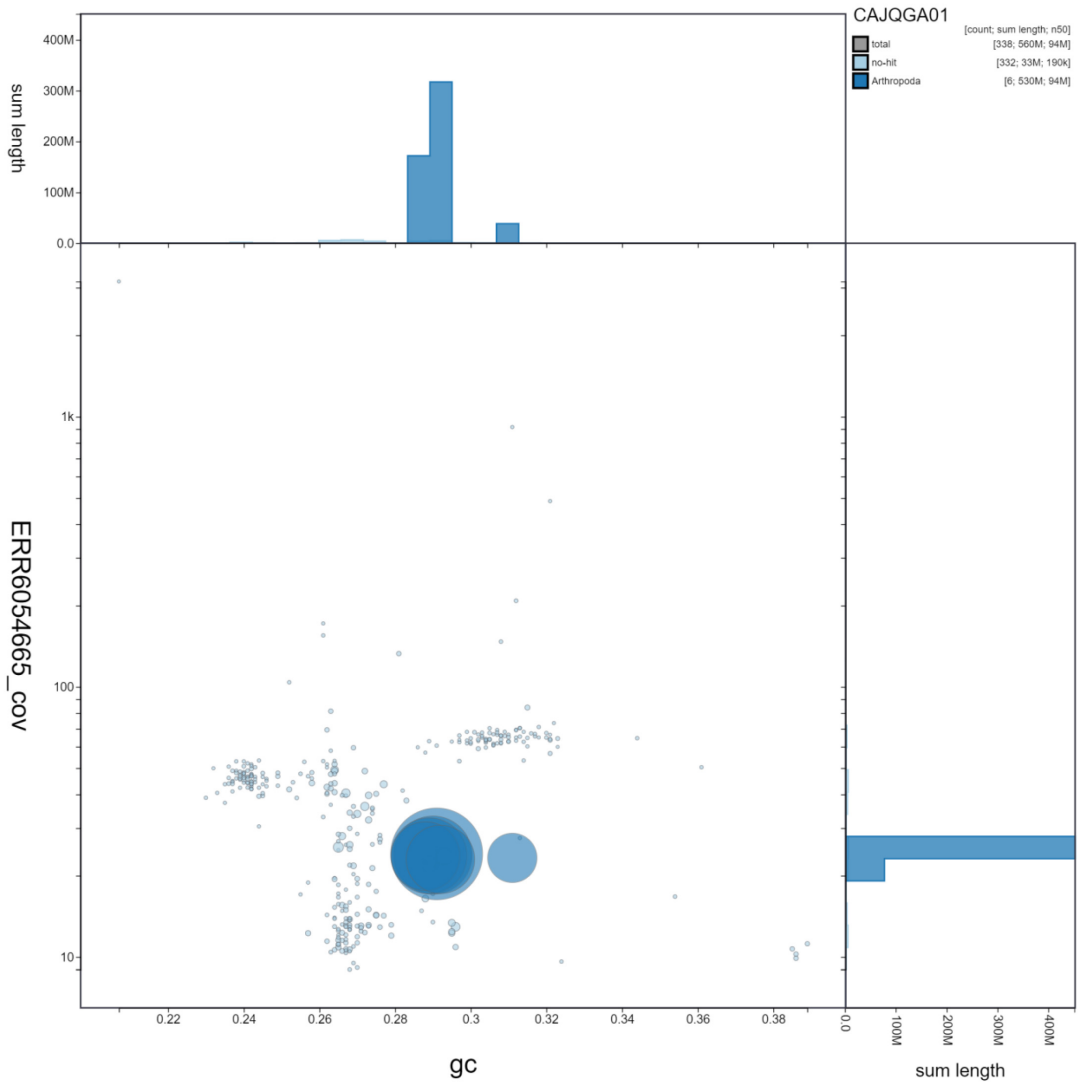
Genome assembly of *Chrysoperla carnea*, inChrCarn1.1: GC coverage. BlobToolKit GC-coverage plot. Scaffolds are coloured by phylum. Circles are sized in proportion to scaffold length. Histograms show the distribution of scaffold length sum along each axis. An interactive version of this figure is available at https://blobtoolkit.genomehubs.org/view/inChrCarn1.1/dataset/CAJQGA01/blob.

**Figure 3.**
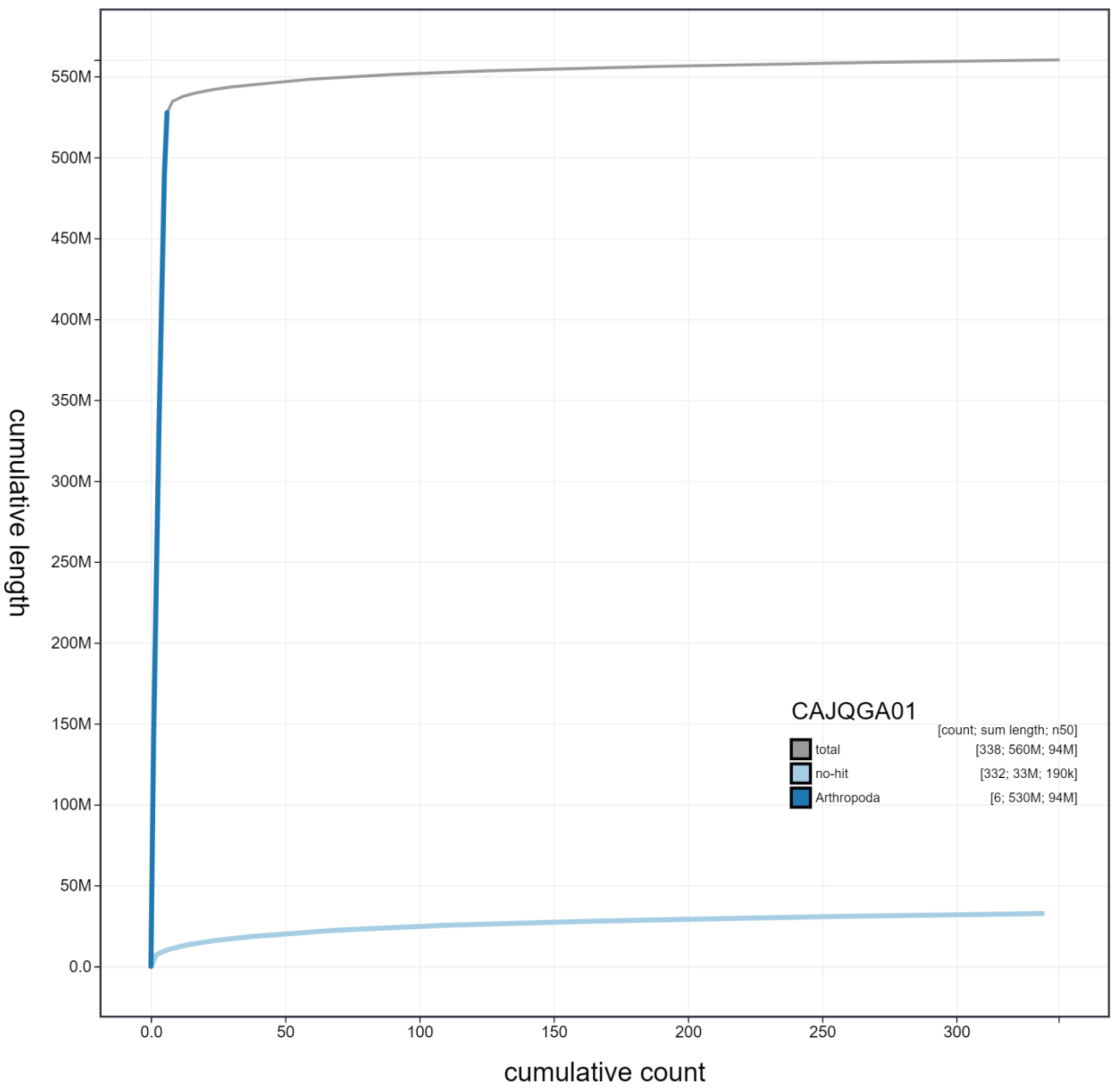
Genome assembly of *Chrysoperla carnea*, inChrCarn1.1: cumulative sequence. BlobToolKit cumulative sequence plot. The grey line shows cumulative length for all scaffolds. Coloured lines show cumulative lengths of scaffolds assigned to each phylum using the buscogenes taxrule. An interactive version of this figure is available at https://blobtoolkit.genomehubs.org/view/inChrCarn1.1/dataset/CAJQGA01/cumulative.

**Figure 4.**
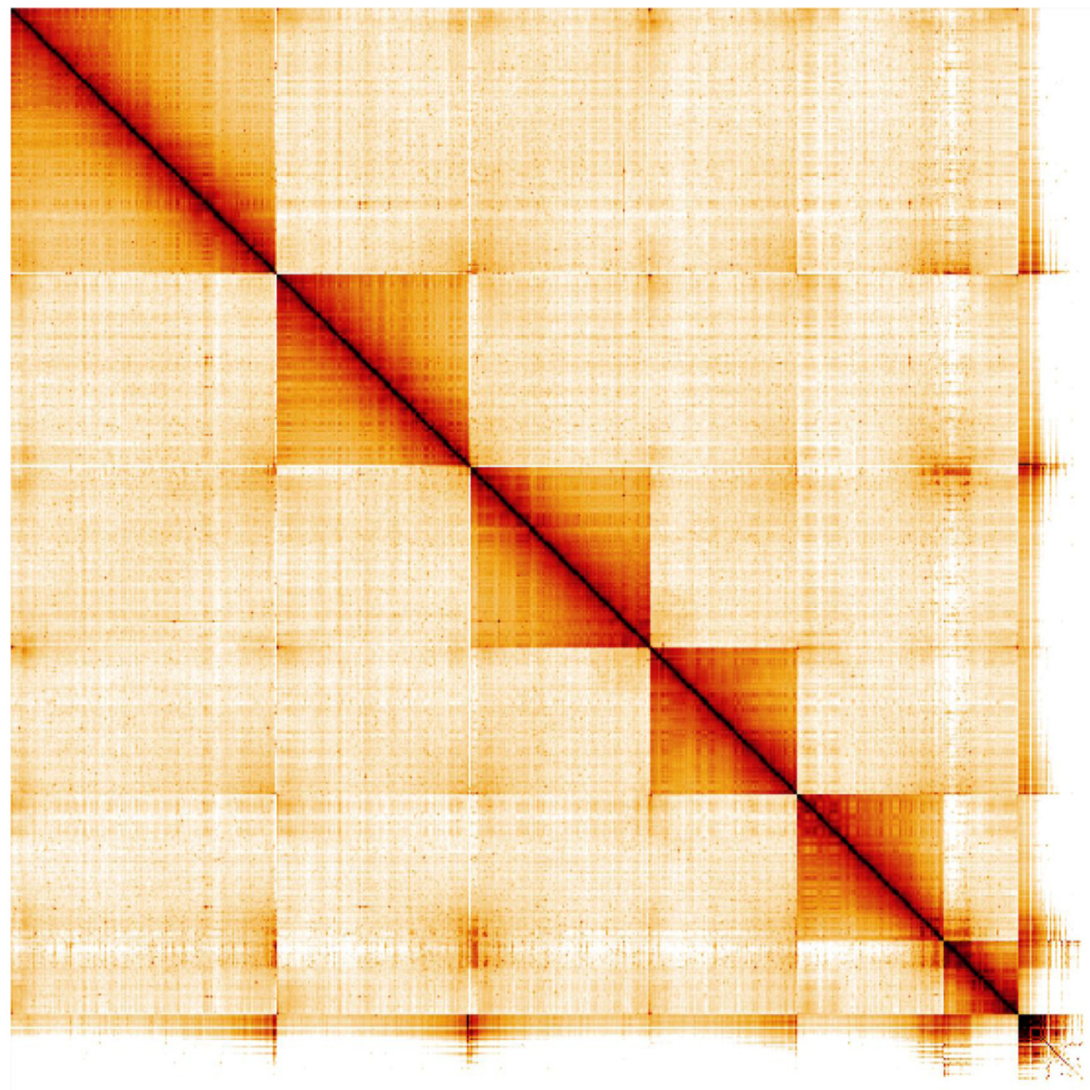
Genome assembly of *Chrysoperla carnea*, inChrCarn1.1: Hi-C contact map. Hi-C contact map of the inChrCarn1.1 assembly, visualised in HiGlass. Chromosomes are shown in order of size from left to right and top to bottom.

**Table 2.** Chromosomal pseudomolecules in the genome assembly of *Chrysoperla carnea*, inChrCarn1.1.

INSDC accession	Chromosome	Size (Mb)	GC%
FR997754.1	1	139.98	29.1
FR997755.1	2	100.74	29
FR997756.1	3	94.41	28.9
FR997757.1	4	77.48	28.8
FR997758.1	5	76.43	29.2
FR997759.1	X	38.62	31.1
FR997760.1	MT	0.02	21.1
-	Unplaced	32.58	27.8

## Genome annotation report

The inChrCarn1.1 genome has been annotated using the NCBI RefSeq annotation pipeline ( Table 1; NCBI *Chrysoperla carnea* Annotation Release 100). The resulting annotation includes 17,649 transcribed mRNAs from 12,985 protein-coding and 2,751 non-coding genes. There is an average of 1.31 transcripts per gene and 4.07 exons per transcript. The annotated genome has a BUSCO v4.1.4 completeness of 96.9% using the endopterygota_odb10 reference set.

## Methods

### Sample acquisition, DNA extraction and sequencing

A single female *C. carnea* was collected from Wytham Woods, Oxfordshire (biological vice-county: Berkshire), UK (latitude 51.772, longitude -1.338) by Liam Crowley, University of Oxford, using a pooter. The sample was identified by the same individual, and preserved on dry ice.

DNA was extracted from the whole organism of inChrCarn1 at the Wellcome Sanger Institute Scientific Operations core from the whole organism using the Qiagen MagAttract HMW DNA kit, according to the manufacturer’s instructions. Pacific Biosciences HiFi circular consensus and 10X Genomics read cloud DNA sequencing libraries were constructed according to the manufacturers’ instructions. Sequencing was performed by the Scientific Operations core at the WSI on Pacific Biosciences SEQUEL II and Illumina HiSeq X instruments. Hi-C data were generated using the Arima v2 Hi-C kit and sequenced on a HiSeq X instrument.

### Genome assembly

Assembly was carried out with Hifiasm ( Cheng *et al*., 2021); haplotypic duplication was identified and removed with purge_dups ( Guan *et al*., 2020). One round of polishing was performed by aligning 10X Genomics read data to the assembly with longranger align, calling variants with freebayes ( Garrison & Marth, 2012). The assembly was then scaffolded with Hi-C data ( Rao *et al*., 2014) using SALSA2 ( Ghurye *et al*., 2019). The assembly was checked for contamination and corrected using the gEVAL system ( Chow *et al*., 2016) as described previously ( Howe *et al*., 2021). Manual curation ( Howe *et al*., 2021) was performed using gEVAL, HiGlass ( Kerpedjiev *et al*., 2018) and Pretext. The mitochondrial genome was assembled using MitoHiFi ( Uliano-Silva *et al*., 2021), which performed annotation using MitoFinder ( Allio *et al*., 2020). The genome was analysed and BUSCO scores generated within the BlobToolKit environment ( Challis *et al*., 2020). Table 3 contains a list of all software tool versions used, where appropriate.

**Table 3.** Software tools used.

Software tool	Version	Source
Hifiasm	0.12	Cheng *et al*., 2021
purge_dups	1.2.3	Guan *et al*., 2020
SALSA2	2.2	Ghurye *et al*., 2019
longranger align	2.2.2	https://support.10xgenomics.com/genome-exome/software/pipelines/latest/advanced/other-pipelines
freebayes	1.3.1-17-gaa2ace8	Garrison & Marth, 2012
MitoHiFi	1.0	Uliano-Silva *et al*., 2021
gEVAL	N/A	Chow *et al*., 2016
HiGlass	1.11.6	Kerpedjiev *et al*., 2018
PretextView	0.1.x	https://github.com/wtsi-hpag/PretextView
BlobToolKit	2.6.2	Challis *et al*., 2020

### Genome annotation

The *C. carnea* assembly was annotated by the NCBI Eukaryotic Genome Annotation Pipeline, an automated pipeline that annotates genes, transcripts and proteins on draft and finished genome assemblies. The annotation was generated from transcripts and proteins retrieved from NCBI Entrez by alignment to the genome assembly, as described here ( Pruitt *et al*., 2014).

### Ethics/compliance issues

The materials that have contributed to this genome note have been supplied by a Darwin Tree of Life Partner. The submission of materials by a Darwin Tree of Life Partner is subject to the Darwin Tree of Life Project Sampling Code of Practice. By agreeing with and signing up to the Sampling Code of Practice, the Darwin Tree of Life Partner agrees they will meet the legal and ethical requirements and standards set out within this document in respect of all samples acquired for, and supplied to, the Darwin Tree of Life Project. Each transfer of samples is further undertaken according to a Research Collaboration Agreement or Material Transfer Agreement entered into by the Darwin Tree of Life Partner, Genome Research Limited (operating as the Wellcome Sanger Institute), and in some circumstances other Darwin Tree of Life collaborators.

## Data availability

European Nucleotide Archive: Chrysoperla carnea (common green lacewing). Accession number PRJEB43807; https://identifiers.org/ena.embl/PRJEB43807.

The genome sequence is released openly for reuse. The *C. carnea* genome sequencing initiative is part of the Darwin Tree of Life (DToL) project. All raw sequence data and the assembly have been deposited in INSDC databases. Raw data and assembly accession identifiers are reported in Table 1.

## Author information

Members of the University of Oxford and Wytham Woods Genome Acquisition Lab are listed here: https://doi.org/10.5281/zenodo.4789929.

Members of the Darwin Tree of Life Barcoding collective are listed here: https://doi.org/10.5281/zenodo.4893704.

Members of the Wellcome Sanger Institute Tree of Life programme collective are listed here: https://doi.org/10.5281/zenodo.5377053.

Members of Wellcome Sanger Institute Scientific Operations: DNA Pipelines collective are listed here: https://doi.org/10.5281/zenodo.4790456.

Members of the Tree of Life Core Informatics collective are listed here: https://doi.org10.5281/zenodo.5013542.

Members of the Darwin Tree of Life Consortium are listed here: https://doi.org/10.5281/zenodo.4783559.
