# Supplementary material for: The genome sequence of the common green lacewing, Chrysoperla carnea (Stephens, 1836)
Source: Wellcome Open Res. 2021 Dec 9;6:334. [Version 1] doi: 10.12688/wellcomeopenres.17455.1 (PMC10116181; doi:10.12688/wellcomeopenres.17455.1)
Supplement: Peer review history

# Peer review: Reviewer response for version 1

**DOI:** 10.21956/wellcomeopenres.19301.r54901

**Published:** 2023-04-19

**Authors:** Emily Hornett

**Affiliations:** 1 University of Liverpool, Liverpool, UK

This article reports the sequencing, assembly and annotation of the genome of a green lacewing, *Chrysoperla carnea*. 

 This article is very concise and clearly written. There are a few comments:

 The Background section would benefit from polishing. The first three lines contain the word "common" three times, and it appears again twice more in this paragraph. A pedantic point, but easily resolved e.g. change "in common with" to "similarly to".

 The Background section would also benefit from two additions: 1) mention of whether lacewing genomes have been sequenced before (they have...just - e.g. Wang et al 2022 - but still underexplored) and 2) why this lacewing is being sequenced and what can we learn from this genome?

 The main text states a male is sequenced, while in the Methods it says female - presumably it was a female sequenced given the lack of a Y chromosome.

 The protocols followed are fairly standard, and the techniques sound. However, the Methods are very brief and there are few parameters described, which makes replication tricky. Given this article type, which is technical in nature, a bit more detail would be useful for readers. 

 For instance, although it is stated that the assembly was checked for contamination, no outcome is noted. Was there contamination? And were any cobionts (non-target organisms) present in the dataset?

 In the DNA extraction section of the Methods, the wording needs clearing up, e.g. "whole organism" is repeated.

 All in all, a useful addition to the genome sequencing project.

Are sufficient details of methods and materials provided to allow replication by others?

Partly

Is the rationale for creating the dataset(s) clearly described?

Partly

Are the datasets clearly presented in a useable and accessible format?

Yes

Are the protocols appropriate and is the work technically sound?

Yes

Reviewer Expertise:

Insect genomics, symbiosis, evolution, microbiomes

I confirm that I have read this submission and believe that I have an appropriate level of expertise to confirm that it is of an acceptable scientific standard, however I have significant reservations, as outlined above.

# Peer review: Reviewer response for version 1

**DOI:** 10.21956/wellcomeopenres.19301.r54904

**Published:** 2023-03-06

**Authors:** Jessica Castellanos-Labarcena

**Affiliations:** 1 University of Guelph, Guelph, Canada

This study presents a genome assembly and annotation for the species *Chrysoperla carnea. *The authors use three sequencing technologies to generate the data and a combination of various software tools to provide an almost complete genome scaffolded into six chromosomal pseudomolecules. The methodology is adequately described for each step, and informative visualizations and tables are included.

 Below you can find my recommendations for the improvement of the document.

1. The abstract and methods sections state that the individual used for the study is female, but the 'Genome sequence report' section states male. This needs to be corrected before indexing.
2. In the 'Background' section, I suggest including a citation for the statement regarding using green lacewing as a biocontrol agent. I also suggest mentioning if any other genome has already been reported for this species complex.
3. In the 'Genome Sequence Report' section could be relevant to mention why the endopterygota_odb10 reference set was used and the effect that has on the results.
4. I suggest fixing the axis labels in Figure 2 (a raw sequence identifier is used in the y-axis; the 'sum length' is inverted) and improving the figure caption. The colours used are very similar and difficult to distinguish when the bubbles overlap. A legend referencing the bubble size to the scaffold length could be beneficial. Also, some of the bars on the histogram are barely visible.
5. In the 'Methods' section, I recommend providing more details about the morphological identification of the specimens. Were pictures taken?
6. In the 'Methods' section, the assembly identifier is used in reference to the species name "DNA was extracted from the whole organism of inChrCarn1…" this should be corrected. The phrase "the whole organism" is repeated twice in this sentence.
7. Figure 4 caption could include more details, for example, of how this figure is generated (visualization of the Hi-C data mapped to sequence data).
8. In the 'Genome Assembly' section, details about some of the parameters used in the software settings should be included when suitable.

Are sufficient details of methods and materials provided to allow replication by others?

Partly

Is the rationale for creating the dataset(s) clearly described?

Yes

Are the datasets clearly presented in a useable and accessible format?

Yes

Are the protocols appropriate and is the work technically sound?

Yes

Reviewer Expertise:

macrogenetics, bioinformatics, genomics.

I confirm that I have read this submission and believe that I have an appropriate level of expertise to confirm that it is of an acceptable scientific standard, however I have significant reservations, as outlined above.

# Peer review: Reviewer response for version 1

**DOI:** 10.21956/wellcomeopenres.19301.r52091

**Published:** 2022-09-16

**Authors:** John Torcivia

**Affiliations:** 1 George Washington University, Washington, DC, USA

The authors present a genome assembly (along with downstream annotation) for a species of lacewing. There is some background provided on the species and usefulness of full assemblies of the genome of any species is helpful to report.

 Protocols used appear to be fairly standard and generally accepted methodologies to perform this assembly and downstream annotation of the genome. While these techniques do not guarantee a perfect assembly, they offer a strong starting point for future research to refine as needed.

 Particular techniques are provided along with supporting papers. One potential improvement to the publication would be additional technical information (which parameters were chosen for each tool for example) to help with potential replication.  Many parameters are related to computation cost but some could have an effect on the actual assembly itself.  That said, it is unlikely that those selected deviate in a significant way from the standard parameters used for these tools and I don't have concern with the general replicability of this dataset. I like the inclusion of a table of the software tools used along with their versions and think this is a helpful addition to these types of articles.

 The sequencing data is uploaded in FASTQ format which is a typical and well understood format. This is deposited into a public repository and available for download and additional utilization. Additional downstream annotation is provided in an NIH database and is represented in generally accepted and understood formats.

Are sufficient details of methods and materials provided to allow replication by others?

Yes

Is the rationale for creating the dataset(s) clearly described?

Yes

Are the datasets clearly presented in a useable and accessible format?

Yes

Are the protocols appropriate and is the work technically sound?

Yes

Reviewer Expertise:

Genomics, cancer genetics, biostatistics, NGS analysis

I confirm that I have read this submission and believe that I have an appropriate level of expertise to confirm that it is of an acceptable scientific standard.
